# Tear film proteome in age-related macular degeneration

**DOI:** 10.1007/s00417-018-3984-y

**Published:** 2018-04-25

**Authors:** Mateusz Winiarczyk, Kai Kaarniranta, Stanisław Winiarczyk, Łukasz Adaszek, Dagmara Winiarczyk, Jerzy Mackiewicz

**Affiliations:** 1 Department of Vitreoretinal Surgery, Medical University of Lublin, Lublin, Poland; 2 Department of Ophthalmology, University of Eastern Finland and Kuopio University Hospital, Kuopio, Finland; 3 Department of Epizootiology, University of Life Sciences of Lublin, Lublin, Poland

**Keywords:** Age-related macular degeneration, Biomarker, Proteomics, Tear film

## Abstract

**Purpose:**

Age-related macular degeneration (AMD) is the main reason for blindness in elderly people in the developed countries. Current screening protocols have limitations in detecting the early signs of retinal degeneration. Therefore, it would be desirable to find novel biomarkers for early detection of AMD. Development of novel biomarkers would help in the prevention, diagnostics, and treatment of AMD. Proteomic analysis of tear film has shown promise in this research area. If an optimal set of biomarkers could be obtained from accessible body fluids, it would represent a reliable way to monitor disease progression and response to novel therapies.

**Methods:**

Tear films were collected on Schirmer strips from a total of 22 patients (8 with wet AMD, 6 with dry AMD, and 8 control individuals). 2D electrophoresis was used to separate tear film proteins prior to their identification with matrix-assisted laser desorption/ionization time of flight spectrometer (MALDI-TOF/TOF) and matching with functional databases.

**Results:**

A total of 342 proteins were identified. Most of them were previously described in various proteomic studies concerning AMD. Shootin-1, histatin-3, fidgetin-like protein 1, SRC kinase signaling inhibitor, Graves disease carrier protein, actin cytoplasmic 1, prolactin-inducible protein 1, and protein S100-A7A were upregulated in the tear film samples isolated from AMD patients and were not previously linked with this disease in any proteomic analysis.

**Conclusion:**

The upregulated proteins supplement our current knowledge of AMD pathogenesis, providing evidence that certain specific proteins are expressed into the tear film in AMD. As far we are aware, this is the first study to have undertaken a comprehensive in-depth analysis of the human tear film proteome in AMD patients.

**Electronic supplementary material:**

The online version of this article (10.1007/s00417-018-3984-y) contains supplementary material, which is available to authorized users.

## Introduction

According to WHO, 285 million people are estimated to be visually impaired worldwide: 39 million are blind and 246 million have poor vision (www.who.int/mediacentre/factsheets/fs282/). AMD is the leading cause of blindness in the elderly population in the Western countries. The latest recent study revealed that early AMD signs can be detected already in patients younger than 30 years of age [1]. Due to the absence of an effective preventive treatment, the number of patients severely disabled by AMD is expected to increase by more than 50% in the next 20 years. The disease not only exerts a tremendous impact on the physical and mental health of the geriatric population and their families but it is also becoming a major public health and financial burden [2].

AMD is characterized by a progressive loss of central vision; this is attributable to degenerative and neovascular changes in the macula, the highly specialized region of the central retina responsible for fine and color vision [3]. AMD is divided into the slowly developing dry (80–85% of cases) and the rapidly blinding wet (15–20%) forms. The prevalence of AMD increases with age; in the age-category of 65–74, its prevalence is 15%; from 75 to 84, it grows to 25%; and in individuals aged 85 and older, the prevalence rises to 30%. A reasonable overall estimate of the incidence of advanced AMD in persons aged 65–74 years is 1%, increasing to 5% in the age-category of 75–84 years, and further up to 13% in subjects 85 years old and above [4]. In Poland, 14,000 new AMD patients are diagnosed each year. AMD has been commonly called “the blindness epidemic,” (http://retinaamd.org.pl/wp-content/uploads/2016/02/Audytraport.pdf).

The etiology of AMD is known to be multifactorial [5]. In addition to a strong genetic component (i.e., defects in certain genes, e.g., ARMS, CFH, C3), and aging, there are thought to be environmental risk factors such as smoking, obesity, hypertension, and hypercholesterolemia which predispose to AMD [4]^.^ The macula area of the retina is a region with a high metabolic rate; it is also subjected to a high oxygen pressure and redox reactions continuously generating highly reactive radicals. It is widely believed that the presence of chronic oxidative stress, impaired autophagy, and inflammation are all strongly linked to the pathogenesis of AMD [3]. The combination of those processes ultimately leads to the death of the RPE cells [6, 7]. One hallmark of AMD is the detrimental accumulation of lysosomal lipofuscin in the RPE cells and the build-up of extracellular drusen deposits between the basal lamina of the RPE and the inner collagenous layer of Bruch’s membrane. High amounts of lipofuscin and drusen deposits predict AMD progression and severity. Choroidal neovascularization, a hallmark of wet AMD, may develop in certain AMD cases. If not treated, these pathological processes promote fibrogliosis, resulting in the production of a disciform scar and severe loss of vision. The available treatment options, such as anti-VEGF intravitreal injections, are useful only in patients suffering from wet AMD and can only delay the progression of the disease [3]. One major public health challenge is to devise an effective primary prevention strategy for identifying high-risk AMD patients and to improve current follow-up as well as discovering more effective treatments. However, in order to achieve these goals, a better understanding of AMD’s pathophysiological details is required.

The primary clinical hallmark of AMD is RPE degeneration. The post-mitotic RPE cells in the macula are constantly exposed to a high metabolic and oxidative stress due to the daily heterophagic processing of the high concentration of polyunsaturated fatty acids (PUFAs) originating from the retinal outer segments, the vigorous choriocapillaris circulation, and the constant exposure to light [3]. During the aging of RPE cells, their capacity to neutralize mitochondrial-derived reactive oxidative species (ROS) diminishes due to a decline in antioxidant production, impaired DNA damage or protein repair processes, and disturbed proteolysis [8, 9]. The intense ROS production and impaired defense systems further damage cellular proteins accentuating the detrimental protein aggregation. The production of lysosomal lipofuscin is one example of the disturbed proteostasis in RPE cells [10–12]. Oxidized retinal outer segment discs are not efficiently digested in the lysosomes of aged RPE cells, instead, they become deposited in the form of lipofuscin. The autofluorescent lipofuscin is a complex heterogeneous mixture of lipid–protein aggregates, which sensitizes RPE cells to light and increases oxidative stress, ultimately evoking misfolding of proteins. The heat-shock protein (Hsp) stress response is the only system capable of refolding misfolded proteins, a crucial mechanism promoting cellular survival in times of oxidative stress [13]. The upregulation of Hsps has been detected in RPE homogenates isolated from human donor AMD samples [14, 15]. This is not only an indication of the over-stressed RPE cells, but it also highlights the dysfunction of the proteasomal clearance [16]. Once Hsps’ repair capacity has become exceeded, single peptide species can be degraded by the proteasome, while larger aggregates are broken down by selective macroautophagy. Starvation, hypoxia, oxidative stress, and protein aggregation are well-known factors for inducing autophagy [3, 17, 18]. Autophagy is a fundamental mechanism for reducing the amount and toxicity of the protein aggregates and to maintain retinoid levels to support vision [17, 18]. However, the autophagy activity is known to be reduced during the aging process of the RPE cells and in AMD [9, 17, 18].

A tear film covers the surface of the eye, providing protection, lubrication, and nutrition. It consists of three layers: the outermost lipid layer, the intermediate aqueous layer, and the innermost mucus layer. The tear film is produced by lacrimal glands and Meibomian glands, accessory glands, and goblet cells [19]. Since it is readily accessible, it has been a target in proteomic programs investigating various ocular and systemic diseases such as dry eye syndrome [20–22], diabetes [23, 24], Parkinson’s disease, multiple sclerosis, or cancer [25].

Previous ocular proteome studies in AMD have been focused on retina, vitreous body, aqueous humor, or blood, with multiple proteins identified in each of them [26–42]. Post-mortem retinal samples show differences between protein distribution between central and peripheral retina in AMD [27], also drusen proteomic composition seems to be different in healthy individuals and AMD patients [40]. Yuan et al. showed elevated levels of metalloproteinase inhibitor 3, vitronectin, C9, clusterin, α-crystallin B, protein-glutamine γ-glutamyltransferase 2, and C3 in the Bruchs membrane-choroid complex obtained from human donors [43].

Ecker et al. identified multiple inflammation-related proteins in the vitreous samples [27]. Matrix metalloproteinase-9 (MMP9) was elevated together with increased subretinal fluid [44]. In other studies by Koss et al. and Nobl et al., where vitreous samples were also obtained, alpha-1-antitrypsin, fibrinogen alpha chain, prostaglandin H2 D-isomerase, opticin, pigment epithelium-derived factor, and prostaglandin-H2 d-isomerase were also linked with AMD pathogenesis [28, 29].

In aqueous humor (AH) analyzed by MRM-MS techniques, plasma protease C1 inhibitor, TGFB1, ceruloplasmin, and pigment-epithelium-derived factor were reported to be significantly different between control and AMD group [31]. Yao et al. reported that the AH protein composition was significantly different between wet AMD and non-AMD patients, with 78 differentiating proteins (68 identified). They proposed crystallins, lipocalin-1, and cyclooxygenase-2 as key players in AMD pathogenesis [30]. Kang et al. selected six proteins as potential biomarkers in AH: actin, myosin-9, Hsp-70, cathepsin D, and cytokeratin 8 and 14 [36]. In more recent study, Ng et al. showed correlation between high angiopoietin-2 levels with disease severity at presentation. Hepatocyte growth factor (HGF), interleukin 8, and TIMP-1 levels were also elevated in AMD group [37]. Interestingly, those findings are in contrary to previous reports of lower angiopoietin 2 levels in AMD patientst [45].

In blood samples obtained from AMD patients, noticeable differences between analyzed groups were also observed. Iannaccone et al. suggested that circulating autoantibodies play a role in AMD pathogenesis, and five of the possible autoreactivity targets were conclusively identified: two members of the heat-shock protein 70 (HSP70) family, HSPA8 and HSPA9; another member of the HSP family, HSPB4, also known as alpha-crystallin A chain (CRYAA); Annexin A5 (ANXA5); and Protein S100-A9, also known as calgranulin B [32, 33]. Kim and et al. in proposed vinculin, phospholipid transfer protein (PLTP), and mannan-binding lectine serine protease 1 (MASP-1) as potential AMD biomarkers, with high AUC values (i.e., 0.936 for PLTP) [38, 41]. Xu et al. identified 28 proteins that were elevated in AMD patients compared to control group [42].

While most of the abovementioned studies propose different AMD biomarkers, proteins identified as differentiating are usually involved in inflammation, impaired autophagy, neovascularization, and oxidative stress response. All of those are well documented as key processes in AMD development. Invasively taken samples are hard to reproduce in the same patients group. This feature reduces their usefulness for patient monitoring and validating the obtained results. Tear film can be taken from the same patient numerous times, even by each appointment.

Our plan was to perform first comprehensive analysis of tear film proteome in AMD patients. To our best knowledge, there is presently no study that would evaluate deeply tears protein composition in AMD. All the other AMD proteomic studies present similar set of expressed proteins, involved in inflammation, microtubule organization, and protein refolding processes. We hypothesize similar set of proteins might be present in tear film.

## Materials and methods

The study was approved by Bioethical Committee of Medical University of Lublin by declaration number KE-0254/238/2015. Informed consent was obtained from every patient enrolled in the study. The purpose and design of the study, as well as its possible complications, were explained to every patient, and written consents were obtained. All experiments followed the provisions of the Declaration of Helsinki.

Patients with wet or dry AMD as well as a control group of cataract patients were included in this single-center prospective study. We applied the following exclusion criteria: eyelid problems (entropion, ectropion, Meibomian Gland Dysfunction and other anterior segment disorders), use of contact lenses, amaurosis, patients suffering from eye trauma, corneal ulcer, uveitis, ophthalmic surgery excluding phacoemulsification, diabetes mellitus. Hypertension was not an exclusion criterion if it did not result in signs of hypertensive retinopathy.

There were 22 patients included in the study; 8 patients with wet AMD, 6 with dry AMD, and 8 were in the control group. Sex distribution was similar between men and women in each group. All criteria-satisfying patients underwent a full ophthalmic examination by the same ophthalmologist consisting of visual acuity test, slit lamp examination, intraocular pressure measurement, optical coherent tomography, and fluorescent angiography if needed.

As ocular surface disease would disturb the results, each patient’s eyelids and anterior segment were carefully assessed. Meibomian Gland Dysfunction was absent in all patients, tear film break-up time (BUT) was within normal limits (over 10 s), and all the patients had Schirmer test result over 13 mm in 5 min.

The wet AMD consisted of eight patients, four men and four women with mean age of 78.1 (SD = 6.73). Mean best corrected visual acuity (BCVA) was 0.25 for right eye (SD = 0.21) and 0.18 for left eye (SD = 0.1) in Snellen charts. Mean intraocular pressure (IOP) was 14.1 for right eye (SD = 3.04) and 14.38 for left eye (SD = 3.29). Schirmer test result in 5 min time was 17.5 mm (SD = 2.39) for right and 17.36 mm (SD = 2.39) for left eye. On slit lamp examination, patients presented active form of AMD in at least one eye with intra/subretinal fluid presence. Seven of them were previously treated with anti-VEGF therapy in one or both eyes, and one patient has undergone a cataract surgery. One patient presented naïve form of wet AMD, with no previous treatment. All the clinical findings were validated by fundus photographs and fluorescein angiography combined with SD-OCT. Environmental risk factors, BMI, smoking, and systemic diseases, were assessed. Average BMI was 26.99 (SD = 2.65) in wet AMD cases. Five patients were active or previous smokers; six were treated for hypertension and two for cardiovascular disease. All the patients are shown in Table 1.

**Table 1:** Wet AMD patients

Sex/age	BCVA OD/OS (Snellen)	IOP (mmHg) OD/OS	Schirmer test result (mm in 5 min)	SRF/CRT(μm)/PED	Ocular interventions	BMI	Smoking (pack-year)	Systemic diseases
M76	0.1/0.1	14/16	20/20	OS—SRF(+), CRT 326, PED(+) OD—macular scar	–	28.1	30	HT, CVD
F76	0.2/CF 0.5m	12/12	18/20	OD—drusen (+++) OS—SRF(+), CRT 330	Anty-VEGF 4× do OS	30.1	–	HT, CVD
F77	0.5/0.2	15/12	20/20	OD-normal OS—SRF(+), CRT 331	Anty-VEGF 2× do OS	27.3	–	HT
M91	0.1/0.2	19/20	14/16	OD—SRF(+), CRT 248 OS—macular scar	Anty-VEGF ODS 5×	22.2	40	–
F77	0.5/0.3	9/11	18/15	OD—normal OS—SRF(+), CRT 421	Anty-VEGF OS 1×	29.5	–	HT
M73	0.1/0.2	13/12	14/14	OD—SRF(+), CRT 230 OS—macular scar	Phaco + ILCP ODS Anty-VEGF ODS 3×	25.4	20	–
M70	CF 1 m/0.1	17/18	19/17	OD—subretinal hemorrhage + CNV OS—macular scar	Anty-VEGF ODS 4×	24.9	30	HT
F85	0.5/0.3	14/14	17/17	OD—drusen(+++) OS—SRF(+), CRT 389	Anty-VEGF OS 3×	28.4	10	HT

The dry AMD group consisted of six patients, three men and three women with mean age of 76.3 (SD = 5.0). Mean best corrected visual acuity (BCVA) was 0.18 for right eye (SD = 0.17) and 0.27 for left eye (SD = 0.12) in Snellen charts. Mean intraocular pressure (IOP) was 12.7 for right eye (SD = 2.6) and 12.2 for left eye (SD = 1.7). Schirmer test result in 5-min time was 17.0 mm (SD = 1.79) for right and 18.33 mm (SD = 1.63) for left eye. On slit lamp examination, they presented either confluent drusen or geographic atrophy. None of the patients has undergone any ocular surgical intervention. All the clinical findings were validated by fundus photographs and SD-OCT. In the dry AMD group, average BMI was 25.6 (SD = 3.15). Two patients were active or previous smokers; four were treated for hypertension and two for cardiovascular disease. All the patients are shown in Table 2.

**Table 2:** Dry AMD patients

Sex/age	BCVA OD/OS (Snellen)	IOP (mmHG) OD/OS	Schirmer test result (mm in 5 min)	Macula condition	Ocular interventions	BMI	Smoking (pack-year)	Systemic diseases
F79	0.1/0.4	11/10	18/19	OD—GA OS—confluent drusen	–	27.8	–	–
M72	0.2/0.4	13/13	20/20	ODS—confluent drusen	–	22.9	40	–
F69	0.4/0.3	11/11	16/20	ODS—confluent drusen	–	24.1	–	HT
F80	0.1/0.2	17/14	16/16	OD—GA OS—confluent drusen	–	21.8	–	HT, CVD
M82	0.2/0.2	14/14	15/17	ODS—confluent drusen	–	29.8	–	HT, CVD
M76	0.1/0.1	10/11	17/18	ODS—GA	–	27.4	30	HT

Control group consisted of eight patients, three men and five women with mean age of 75 (SD = 3.0). Mean best corrected visual acuity (BCVA) was 0.6 for right eye (SD = 0.3) and 0.59 for left eye (SD = 0.26) in Snellen charts. Mean intraocular pressure (IOP) was 13.13 for right eye (SD = 3.23) and 12.86 for left eye (SD = 1.73). Schirmer test result in 5-min time was 16.63 mm (SD = 2.20) for right and 17.0 mm (SD = 2.20) for left eye. On slit lamp examination, there were no abnormalities in either the anterior or posterior segment. The control group was recruited from the patients qualified for standard cataract surgery. Three of those patients were pseudophakic, with surgery performed at least 1 year ago. Posterior pole was evaluated by fundus photograph and SD-OCT. In the control group, average BMI was 27.4 (SD = 2.6). Five patients were active or previous smokers; five were treated for hypertension and three for cardiovascular disease. All the patients are shown in Table 3.

**Table 3:** Control group patients

Sex/age	Visus BCVA OD/OS	IOP (mmHG)	Schirmer test result (mm in 5 min)	Ocular interventions	BMI	Smoking (pack-years)	Systemic diseases
M80	1.0/0.8	12/12	13/16	Phaco + ILCP OD	23.5	35	HT, CVD
F74	0.6/0.7	12/13	20/19	–	28.1	–	HT,CVD
F73	0.9/0.6	16/13	17/18	–	30.4	–	HT
M77	0.9/0.5	10/12	15/14	Phaco + ILCP OD	29.1	80	HT
F74	0.5/0.6	15/14	18/20	–	28.9	20	HT
F73	0.2/0.2	19/16	17/14	–	29.4	10	–
F71	0.3/1.0	10/10	18/18	Phaco + ILCP OS	24.2	–	CVD
M78	0.4/0.3	11/13	15/17	–	25.6	13	–

Tear film was collected from each eye onto a Schirmer strip (TearFlo, HUB Pharmaceuticals LLC [46, 47]. Each collection was performed by the author, in the morning hours, between 8 and 11 a.m. If fluorescein angiography was performed, material was collected always beforehand. Sterile gloves were always used by the investigator. Schirmer strips were placed into the lower sacs of both eyes at 1/3 of the distance of the eyelid from the nasal canthus without anesthesia. This method was chosen over a microcapillary, flush tear, or basal tear collection mainly due to the patient comfort. As a standard clinical procedure, it is usually well known and tolerated by the patients. There is currently no consensus about which method of collection should be used for proteomic analysis [48–51]. After holding the strips in place for 5 min, they were removed, transferred to a 1.5-mL Eppendorf tube without any buffer, and were then immediately frozen at − 80 °C. In the next step, the proteins were eluted from the strips into 8 M urea buffer containing 3% CHAPS detergent and 25 mM dithiothreitol (DTT), 30 μg of protease inhibitor cocktail, after which the samples were desalted and concentrated through Amicon® Ultra 0.5-mL centrifugal filters 3 kDa (Merck). The protein concentration was determined in a Direct Detect® infrared spectrometer (Merck).

In the 2D electrophoresis, the sample buffer (Bio-Rad) was added to a sample containing of 40 μg total protein. Next, the obtained solution was placed with the immobilized pH gradient (IPG) strip (17 cm, pH 3–10, linear pH gradient, Bio-Rad) in a rehydrating plate for isoelectric focusing with the Hoefer IEF100 instrument. The IPG strips were incubated for 15 min in equilibration buffer (50 mM Tris–HCl, pH 8.8, 6 M urea, 30% glycerol, 2% SDS) containing 1% DTT, after which the incubation was continued for 15 min in equilibration buffer containing 2.5% iodoacetamide. The second electrophoretic separation was conducted using 12.5% polyacrylamide gel in BIO-RAD PROTEAN II xi Cell. Vertical separation was achieved with 600 V/50 mA/30 W in 0.025 M Tris/GLY pH 8.3 buffer. After electrophoretic separation, proteins were silver stained according to Shevchenko et al. [52]. Initial quantitative and qualitative analyses were performed by comparing the electrophoretic gels from all the groups.

All spots were cut out from the 2D electrophoresis gels, washed, and destained for proteomic analysis. The subsequent reduction and alkylation processes were applied using dithiothreitol and iodoacetamide, respectively. After dehydration, gel pieces were incubated with trypsin in 50 mM ammonium bicarbonate for overnight digestion at 37 °C (Promega, Trypsin Gold, Mass Spectrometry Grade, Technical Bulletin). After digestion, the peptides were extracted three times with 50 μl of a solution containing ACN/H2O/TFA (500:450:50) with sonification for 15 min at room temperature in an ultrasonic water bath. The supernatant was collected and dried in the CentriVap (Labconco). Concentrated and purified peptides were obtained by employing μC18 Zip-TIP (PR 02358, Merck Technical Note) and finally transferred onto the MTP plate (Bruker).

MALDI-TOF was used as a soft ionization method because it only introduces a charge and does not cause fragmentation of the analyzed compound. The experiment was conducted in an ultrafleXtreme (Bruker) machine with a TOF/TOF detector to guarantee high accuracy and resolution of the measurements. All of the spectra were collected within the 900–4000 Da range in the active reflection mode, and this mass range was used to acquire the tandem mass spectrometry (MS/MS) spectra. HCCA (alpha-cyano-4-hydroxycinnamic acid, portioned; Bruker) was used as the matrix in the dried droplet method (0.5 μl sample + 0.5 μl matrix) following the standard manufacturer’s protocol for peptide analysis. An MTP AnchorChip 384 (Bruker) with hydrophilic spots was used as the holder for sample preparation. Each sample was spotted onto three different active spots, and the profiled spectra were calibrated using the peptide mixture Peptide Calibration Standard I (Bruker). The flexControl program 3.3 (version 108) was used for mass spectra collection, flexAnalysis 3.3 (version 80) was used for analysis, and finally, the SwissProt database was searched using the software BioTools 3.2 (version 4.48). All spectra were systematically processed as follows: smoothing performed by the Savitsky-Golay method, baseline subtraction was performed by the Top Hat baseline algorithm, peak geometry was characterized by the Stanford Network Analysis Platform (SNAP) algorithm, and all peaks with a signal ratio above 4 were qualified for further analysis. The parameters for the Mascot database search as follows: errors in both MS and MS/MS mode at 0.3 D38, global modification of carbamidomethyl (C), possible modification and oxidation (M)14, partials at 1, and trypsin enzyme. Spectra with peptide matches above 5 peaks were considered statistically significant, and only five proteins were identified with a single peptide match. All of the peptide mass fingerprint spectra were analyzed again in MS/MS mode to confirm their exact amino acid sequence.

## Results

**Fig. 1:**
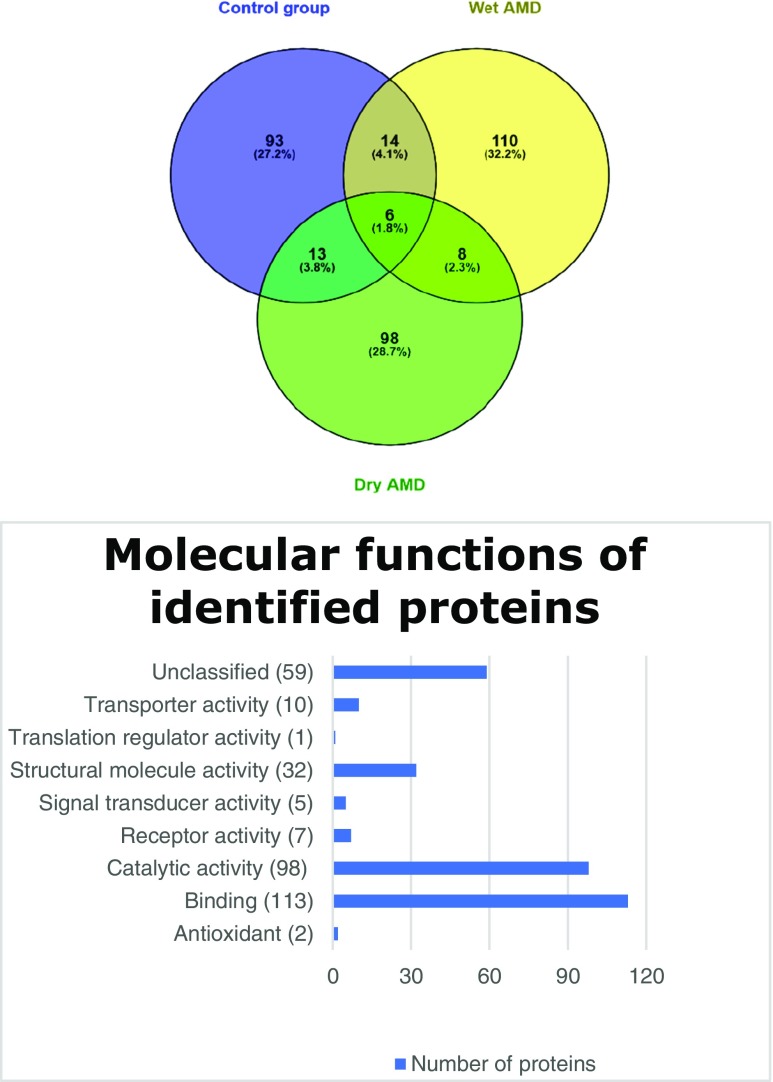
The upper figure represents the number of identified proteins with their distribution and the overlap between the analyzed groups. The lower chart describes the molecular function of all identified proteins based on http://pantherdb.org gene ontology online tool

The tear film proteome reveals specific proteins that can be linked with AMD pathology. We analyzed all of the proteins cut from the electrophoretic gels and created a tear film proteome map for each study group. A total of 342 proteins were identified: 138 for wet AMD, 125 for dry AMD, and 126 for control group patients (Fig. 1). In our study, eight proteins were recognized exclusively for AMD, i.e., in the both wet and dry disease forms: shootin-1, histatin-3, fidgetin-like protein 1, SRC kinase signaling inhibitor, Graves disease carrier protein, actin cytoplasmic 1, prolactin-inducible protein 1, and protein S100-A7A. All of the proteins identified are shown in the supplementary material Tables 1, 2, 3 with obtained score, mass, matching number, and UniProt accession number for control group, wet AMD, and dry AMD, respectively. Fig. 1 shows the Venn diagram of all proteins identified and reveals how they overlap in different study groups according to Venny 2.0.1 application (Oliveros). The obtained data was analyzed using the PANTHER classification system (http://pantherdb.org) to obtain a functional classification with a special focus on metabolic pathways. Table 4 shows proteins for wet AMD and Table 5 for dry AMD. The wet AMD proteomic results reveal that there was an upregulation of proteins that regulate inflammation and neovascularization. Additionally, with respect to the growing understanding of importance of clearance mechanisms in AMD etiology, we added Hsp90-related proteins found in tear film, based on the Picard laboratory database which is available online (https://www.picard.ch/downloads/Hsp90interactors.pdf) (Table 6). We observed that 17 of the analyzed proteins had been previously recognized as potential AMD biomarkers, i.e., they had been detected in aqueous humor, vitreous, blood plasma, central and peripheral retina, or drusen (Table 7).

**Table 4:** Proteins specific for wet AMD with related pathways

Protein	Pathway (by PANTHER classification)
*Angiogenesis related*	
Ras GTPase-activating protein 1 (RASA1) Signal transducer and activator of transcription 3 (STAT3)	PDGF signaling pathway
Fibroblast growth factor receptor 1 (FGFR1) Ras GTPase-activating protein 1 (RASA1) Signal transducer and activator of transcription 3 (STAT3)	Angiogenesis
Fibroblast growth factor receptor 1 (FGFR1) Ras GTPase-activating protein 1 (RASA1)	FGF signaling pathway
Calcium/calmodulin-dependent 3′,5′-cyclic nucleotide phosphodiesterase 1A (PDE1A) Signal transducer and activator of transcription 3 (STAT3)	CCKR signaling map
Ras GTPase-activating protein 1 (RASA1) Signal transducer and activator of transcription 3 (STAT3)	EGF receptor signaling pathway
Signal transducer and activator of transcription 3 (STAT3)	Gonadotropin-releasing hormone receptor pathway
*Apoptosis related*	
Insulin-like growth factor-binding protein 3 (IGFBP3) Cyclin-G1 (CCNG1) Cyclin-dependent kinase 4 inhibitor D (CDKN2D)	p53 pathway
Cyclin-G1 (CCNG1)	p53 pathway feedback loops 2
Bcl-2-related ovarian killer protein (BOK) Bcl-2-like protein 10 (BCL2L10)	Apoptosis signaling pathway
*Trafficking*	
Synaptotagmin-like protein 3 (SYTL3)	Synaptic vesicle trafficking
*Inflammation*	
Myosin-13 (MYH13) Signal transducer and activator of transcription 3 (STAT3)	Inflammation mediated by chemokine and cytokine signaling pathway
Ras GTPase-activating protein 1 (RASA1) Signal transducer and activator of transcription 3 (STAT3)	Interleukin signaling pathway
Signal transducer and activator of transcription 3 (STAT3)	JAK/STAT signaling pathway
*Cytoskeleton and cell motility*	
Myosin-13 (MYH13)	Cytoskeletal regulation by Rho GTPase
Collagen alpha-1(IX) chain (COL9A1)	Integrin signaling pathway
*Other*	
Ras GTPase-activating protein 1 (RASA1)	Insulin/IGF pathway-mitogen activated protein kinase kinase/MAP kinase cascade
Signal transducer and activator of transcription 3 (STAT3)	Ras Pathway
Myosin-13 (MYH13)	Nicotinic acetylcholine receptor signaling pathway
Rod cGMP-specific 3′,5′-cyclic phosphodiesterase subunit beta (PDE6B)	Heterotrimeric G-protein signaling pathway-rod outer segment phototransduction
Phosphorylase b kinase regulatory subunit beta (PHKB)	Heterotrimeric G-protein signaling pathway-Gi alpha and Gs alpha-mediated pathway
Retinal dehydrogenase 1 (ALDH1A1)	5-hydroxytryptamine degradation
Myosin-13 (MYH13)	Wnt signaling pathway

**Table 5:** Proteins specific for dry AMD with related pathways

Protein	Pathway (by PANTHER classification)
*Oxidative stress*, *inflammation*, *and proteolysis related*	
Guanine nucleotide-binding protein subunit alpha-11 (GNA11)	Inflammation mediated by chemokine and cytokine signaling pathway
Myc proto-oncogene protein (MYC)	Interleukin signaling pathway
Ribosomal protein S6 kinase alpha-3 (RPS6KA3)	
Myc proto-oncogene protein (MYC)	Oxidative stress response
Dual specificity protein phosphatase 22 (DUSP22)	
Guanine nucleotide-binding protein subunit alpha-11 (GNA11)	Histamine H1 receptor-mediated signaling pathway
Guanine nucleotide-binding protein subunit alpha-11 (GNA11)	5HT2 type receptor-mediated signaling pathway
Guanine nucleotide-binding protein subunit alpha-11 (GNA11)	Alpha adrenergic receptor signaling pathway
Myc proto-oncogene protein (MYC)	CCKR signaling map
Ribosomal protein S6 kinase alpha-3 (RPS6KA3)	
Guanine nucleotide-binding protein subunit alpha-11 (GNA11)	Corticotropin releasing factor receptor signaling pathway
cGMP-dependent protein kinase 2 (PRKG2)	Endothelin signaling pathway
Endothelin-converting enzyme 1 (ECE1)	
Guanine nucleotide-binding protein subunit alpha-11 (GNA11)	
Fibroblast growth factor 9 (FGF9)	FGF signaling pathway
Pyruvate kinase PKM (PKM)	Glycolysis
5′-AMP-activated protein kinase subunit gamma-2 (PRKAG2)	Gonadotropin-releasing hormone receptor pathway
Guanine nucleotide-binding protein subunit alpha-11 (GNA11)	
F-box/WD repeat-containing protein 1A (BTRC)	Hedgehog signaling pathway
Guanine nucleotide-binding protein subunit alpha-11 (GNA11)	Heterotrimeric G-protein signaling pathway-Gq alpha and Go alpha-mediated pathway
Calpain-7 (CAPN7)	Huntington disease
AP-2 complex subunit alpha-1 (AP2A1)	
Cytoplasmic dynein 1 light intermediate chain 1 (DYNC1LI1)	
Ribosomal protein S6 kinase alpha-3 (RPS6KA3)	Insulin/IGF pathway-mitogen-activated protein kinase kinase/MAP kinase cascade
Laminin subunit alpha-3 (LAMA3)	Integrin signaling pathway
Glutamate receptor 2 (GRIA2)	Ionotropic glutamate receptor pathway
Guanine nucleotide-binding protein subunit alpha-11 (GNA11)	Metabotropic glutamate receptor group I pathway
Glutamate receptor 2 (GRIA2)	Metabotropic glutamate receptor group III pathway
Guanine nucleotide-binding protein subunit alpha-11 (GNA11)	Muscarinic acetylcholine receptor 1 and 3 signaling pathway
Guanine nucleotide-binding protein subunit alpha-11 (GNA11)	Oxytocin receptor-mediated signaling pathway
Myc proto-oncogene protein (MYC)	PDGF signaling pathway
Ribosomal protein S6 kinase alpha-3 (RPS6KA3)	
Guanine nucleotide-binding protein subunit alpha-11 (GNA11)	PI3 kinase pathway
Ubiquitin/ISG15-conjugating enzyme E2 L6 (UBE2L6)	Parkinson disease
Pyruvate kinase PKM (PKM)	Pyruvate metabolism
Ribosomal protein S6 kinase alpha-3 (RPS6KA3)	Ras pathway
Guanine nucleotide-binding protein subunit alpha-11 (GNA11)	Thyrotropin-releasing hormone receptor signaling pathway
F-box/WD repeat-containing protein 1A (BTRC)	Toll pathway-drosophila
Ubiquitin/ISG15-conjugating enzyme E2 L6 (UBE2L6)	Ubiquitin proteasome pathway
Vitamin D-binding protein (GC)	Vitamin D metabolism and pathway
Myc proto-oncogene protein (MYC)	Wnt signaling pathway
F-box/WD repeat-containing protein 1A (BTRC)	
Guanine nucleotide-binding protein subunit alpha-11 (GNA11)	
Myc proto-oncogene protein (MYC)	p53 pathway feedback loops 2

**Table 6:** Proteins linked to Hsp90 according to Picard Laboratory

*Protein*	*Wet AMD*	*Dry AMD*	*Control*
Annexin A3	+	–	–
Myosin-13	+	–	–
Insulin-like growth factor-binding protein 3	+	–	–
Hsp90 co-chaperone Cdc37-like 1	–	+	–
SRC kinase signaling inhibitor	+	+	–
Rho GTPase-activating protein 24	+	+	–
Prolactin-inducible protein	+	+	
β-Actin	+	+	–
Apolipoprotein A	+	–	+
Histones H2B	+	–	+

**Table 7:** Proteins identified as a potential biomarkers in different studies

*Proteins previously identified*	*Wet AMD group*	*Dry AMD group*	*Article no*.
Serum albumin	+	+	1, 5, 6
Apolipoprotein E	Apolipoprotein L6	–	1
Actin beta (1)	Actin cytoplasmic 1	Actin cytoplasmic 1	1, 2
Actin, aortic smooth muscle (2)			
Annexin II	Annexin A3	–	1
Histone H2B C; H2Ao; H2A2	Histone H1.4; H2A type 2-B	–	1
Cystatin C	Cystatin S; SN	Cystatin S	2
Leucine-rich repeat-containing protein 15	–	Leucine-rich repeat-containing protein 16A	2
Pyruvate kinase	–	+	3
HLA-drb1–5	HLA Class I histocompatibility antigen Cw-15 alpha chain		4
Fibroblast growth factor 6	Fibroblast growth factor 8	Fibroblast growth factor 9	4
Peroxiredoxin 3 isoform β	Peroxiredoxin 6	Peroxiredoxin 1	4
NADH dehydrogenase 1α	NADH dehydrogenase 1α (4× subcomplex/subunit)	NADH dehydrogenase 1α (2× subcomplex/subunit)	4
Apolipoprotein a-i; a-iv	Apolipoprotein L6	–	4, 6
Ubiqutin-conjugating enzyme e2	–	Ubiquitin/ISG15-conjugating enzyme E2 L6	4
Vitamin D-binding protein	–	+	4
Lipocalin-1	+	–	5
Serotransferrin	+	–	5, 6

*1* Drusen proteome analysis: an approach to the etiology of age-related macular degeneration. Crabb et al. Proc Natl Acad Sci USA. 2002 Nov 12;99(23):14682–7, *2* Proteomic analysis of the aqueous humor in age-related macular degeneration (AMD) patients. Kim et al. J Proteome Res. 2012 Aug 3;11(8):4034–43, *3* Proteomics of the retinal pigment epithelium reveals altered protein expression at progressive stages of AMD. Nordgaard et al. Invest Ophthalmol Vis Sci. 2006 Mar;47(3):815–22, *4* Comparative proteomic analysis of plasma proteins in patients with age-related macular degeneration. Xu et al. Int J Ophthalmol. 2014 Apr 18;7(2):256–63, *5* Proteomic analysis of the aqueous humor in patients with wet age-related macular degeneration. Yao et al. Proteomics Clin Appl. 2013 Aug;7(7–8):550–60, *6* Proteomics of vitreous humor of patients with exudative age-related macular degeneration. Koss et al. PLoS One. 2014 May 14;9(5):e96895

## Discussion

A total of 110 specific proteins were identified in the wet AMD samples. Interestingly, many of the discovered proteins are known to be involved in the regulation of phagocytosis, inflammation, proteolysis, or alternatively they play a key role in cytoskeletal function. Choroidal neovascularization is one of the hallmarks of wet AMD. New vessels sprout from the choroidal capillaries through Bruch’s membrane into the sub-RPE space or into the retinal layers. The weakened structure of new vascular tissue causes leakage into the retinal layers. This promotes fibrogliosis, resulting in the formation of a disciform scar and severe visual loss if not treated with intravitreal anti-VEGF drugs. The upregulation of several proteins, e.g., VEGF, MMPs, IGF-1, has been linked to the pathology of neovascularization. We detected four tear film proteins involved in several angiogenesis-related pathways, i.e., the PDGF signaling pathway, the EGF signaling pathway, and the CCKR signaling pathway (also linked with cancer [53]) in wet AMD samples [54–56].

Inflammation is one of the best documented processes underlying the pathogenesis of AMD [57–59]. It is strongly associated with chronic oxidative stress and impairment of autophagy [3]. Short-lasting inflammation is beneficial in host defense in a process called para-inflammation, but chronic inflammation leads to the detrimental tissue alterations encountered in AMD and other age-related diseases [60, 61]. We observed upregulation of several inflammatory agents, e.g., RASA1, STAT3, and Myosin-13 in the tear film samples isolated from wet AMD patients. STAT3 has been demonstrated to enhance choroidal neovascularization, and this factor is known to be activated by elevated IL-10 levels [62]. STAT3 has been postulated to be a potential biomarker for diagnosis and treatment of AMD due to its regulatory role in the survival of RPE cells; it is also recognized as playing a role in the regulation of visual cycle and the inflammatory response [63].

RPE cell apoptosis is thought to be an important cell death mechanism in AMD [6]. It consists of two main pathways: the FasL-related extrinsic (death receptor) pathway and the intrinsic (mitochondrial) pathway [64]. Evidence has been obtained of enhanced apoptosis in post-mortem wet AMD samples [65]. Increased apoptosis was also observed in Ccl2^−/−^/Cx3cr1^−/−^ (DKO) mice as well as in ARPE-19 cells exposed to different AMD-like stresses [7]. Interestingly, we observed upregulation of five proteins involved in three apoptosis-related pathways in tear samples isolated from our wet AMD patients. Taken together, it seems that the upregulated proteins involved in inflammation and choroidal neovascularization, as well as in apoptotic processes in RPE cells, can be detected in the tear film of wet AMD patients.

Dry AMD patients showed 97 specific proteins related to 44 pathway patterns. Dry AMD-related proteins were involved in more pathways than those identified in the wet AMD. The two most widely expressed pathways were the Wnt signaling pathway and the Huntington disease pathway. It is noteworthy that there was a major representation of proteins involved in oxidative stress, inflammation, and proteolysis, e.g., the autophagy-related PI3K pathway. Autophagy failure has been reported to be associated with AMD development [6, 18, 66].

AMD is an example of a protein misfolding disease [3]. Heat-shock proteins (Hsps) attempt to refold misfolded proteins and restore protein homeostasis in RPE cells. Hsp90 is a member of the Hsp family that has many interacting targets within cells [67] (https://www.picard.ch/downloads/Hsp90interactors.pdf). In our study, 10 proteins were associated with Hsp90; 3 of them was present only in wet AMD, 1 only in dry AMD, 4 in both forms of AMD, and 2 in both controls and wet AMD. This considerable number of Hsp90-related proteins emphasizes its active involvement in both types of AMD and thus may represent a possible drug target. Hsp90 has been linked to autophagy, a process known to decline in the late stage of AMD [3, 64, 65, 68, 69]. Its role in Alzheimer’s disease [67] and cancer [70] has also been documented. In retina, a short-term inhibition of Hsp90 was demonstrated to promote the viability of cells and improve visual function [71], whereas its prolonged inhibition led to the degradation of its client proteins which may impair visual function [66]. Two Hsp90 client proteins—insulin-like growth factor-binding protein 3 and Annexin A3—have been closely related to the process of choroidal neovascularization [72].

We believe that this is the first comprehensive in-depth analysis of human tear film proteome in patients suffering from different types of AMD. It seems that certain specific proteins are expressed in the tear film, and those biomarkers can be linked with AMD pathology. Our study supports the current view of AMD as a disease where inflammation, oxidative stress, and impaired autophagy play crucial role, making those processes a target for potential future treatment. Due to limitations of this pilot study, further investigations on tear film in AMD should be conducted, involving larger patient groups and division into AMD subtypes.

## Electronic supplementary material

ESM 1 (PDF 75 kb)

ESM 2 (PDF 87 kb)

ESM 3 (PDF 62 kb)
