# Supplementary material for: Tear film proteome in age-related macular degeneration
Source: Graefes Arch Clin Exp Ophthalmol. 2018 Apr 25;256(6):1127–39. doi: 10.1007/s00417-018-3984-y (PMC5956098; doi:10.1007/s00417-018-3984-y)
Supplement: Supplementary file 1 — (PDF 75 kb) [file 417_2018_3984_MOESM1_ESM.pdf]

Tear film proteome in age-related macular degeneration.

Mateusz Winiarczyk[1], Kai Kaarniranta[2], Stanisław Winiarczyk[3], Łukasz Adaszek[3], Dagmara Winiarczyk[3], Jerzy Mackiewicz*[1]

1. Department of Vitreoretinal Surgery, Medical University of Lublin
2. Department of Ophthalmology, University of Eastern Finland and Kuopio University Hospital, Kuopio, Finland
3. Department of Epizootiology, University of Life Sciences of Lublin

*jerzymackiewicz@umlub.pl, 20-079 Lublin, ul. Chmielna 1, Poland, tel./fax: +48 81 53 40 251

Supplementary table 1 - Control group patients identified proteins

Protein name	Score	Mass	Matches	Access no.
**Serum albumin**	210	71.3	9	P02768
**Lactotransferrin**	116	80	17	P02788
**Lipocalin-1**	85	19.4	10	P31025
**SUMO-conjugating enzyme UBC9**	72	18.2	8	P63279
**Ras GTPase-activating protein 2**	70	97.9	12	Q15283
**Integrator complex subunit 6**	66	101	13	Q9UL03
**Zinc finger protein 391**	65	41.8	9	Q9UJN7
**Grainyhead-like protein 3 homolog**	64	71	12	Q8TE85
**Zinc finger protein 624**	64	102.5	12	Q9P2J8
**28S ribosomal protein S22, mitochondrial**	64	41.4	7	P82650
**Putative uncharacterized protein SSBP3-AS1**	62	11	5	Q7Z2R9
**Low-density lipoprotein receptor-related protein 4**	62	217.3	21	O75096
**B-cell antigen receptor complex-associated protein beta chain**	61	26.3	6	P40259
**Telomerase reverse transcriptase**	61	128.6	20	O14746

Protein name	Score	Mass	Matches	Access no.
**Potassium voltage-gated channel subfamily B member 1**	61	96.7	10	Q14721
**Ras and Rab interactor 2**	60	101.1	12	Q8WYP3
**Iron-sulfur protein NUBPL**	60	34.3	7	Q8TB37
**Testis-expressed sequence 33 protein**	60	30.8	9	O43247
**Metallophosphoesterase 1**	60	45.7	7	Q53F39
**Melanoma inhibitory activity protein 3**	60	214.3	17	Q5JRA6
**Protein-glutamine gamma-glutamyltransferase 6**	60	80.1	14	O95932
**Vimentin**	59	53.7	18	P08670
**Protoporphyrinogen oxidase**	59	51.2	7	P50336
**T-complex protein 1 subunit gamma**	59	61.1	10	P49368
**ATP synthase subunit alpha, mitochondrial**	59	59.8	10	P25705
**G-protein coupled receptor 87**	58	42	6	Q9BY21
**Cystatin-S**	58	16.5	5	P01036
**Carbonyl reductase [NADPH] 3**	58	31.2	7	O75828
**Zinc finger protein 585A**	58	90.5	20	Q6P3V2
**Desmin**	58	53.6	5	P17661
**NAD-dependent protein deacetylase sirtuin-3, mitochondrial**	57	43.9	7	Q9NTG7
**Protein phosphatase 1 regulatory subunit 3B**	57	33.1	5	Q86XI6
**HLA class II histocompatibility antigen, DRB1-4 beta chain**	57	30.4	7	P13760
**Signal-regulatory protein beta-1**	57	43.6	10	O00241
**Aurora kinase C**	57	35.9	12	Q9UQB9
**Glucosamine-6-phosphate isomerase 2**	57	31.3	7	Q8TDQ7

Protein name	Score	Mass	Matches	Access no.
**Tetratricopeptide repeat protein 39B**	57	77.9	7	Q5VTQ0
**EH domain-containing protein**	56	61.4	8	Q9H223
**Bromodomain testis-specific protein**	56	108.5	10	Q58F21
**Gamma-aminobutyric acid receptor subunit epsilon**	56	58.9	6	P78334
**Dual specificity protein kinase CLK3**	56	74.3	8	P49761
**N-acetyltransferase ESCO2**	56	69.2	9	Q56NI9
**Suppressor of tumorigenicity 20 protein**	56	9.2	6	Q9HBF5
**Zinc finger protein 780A**	56	76.6	9	O75290
**Interferon-induced protein with tetratricopeptide repeats 3**	56	56.7	8	O14879
**Beta-defensin 107**	55	8.1	5	Q8IZN7
**Protein Njmu-R1**	55	45.3	8	Q9HAS0
**Tudor domain-containing protein 3**	55	73.4	7	Q9H7E2
**Tyrosine-protein kinase Fer**	54	95.2	14	P16591
**Phosphoserine phosphatase**	54	25.2	13	P78330
**FUN14 domain-containing protein 2**	54	20.7	15	Q9BWH2
**IQ and AAA domain-containing protein 1**	54	95.6	12	Q86XH1
**mRNA export factor**	53	41.6	7	P78406
**Cyclin-I**	53	43.2	7	Q14094
**RILP-like protein 1**	53	47.1	11	Q5EBL4
**Thymidine kinase, cytosolic**	53	26.1	7	P04183
**Prolyl 4-hydroxylase subunit alpha-3**	53	61.4	8	Q7Z4N8
**Chondroitin sulfate N-acetylgalactosaminyltransferase 1**	53	61.8	11	Q8TDX6

Protein name	Score	Mass	Matches	Access no.
**Beta-1,3-galactosyl-O-glycosyl-glycoprotein beta-1,6-N-acetylglucosaminyltransferase 3**	52	51.6	12	O95395
**Uncharacterized protein C2orf54**	52	49.9	10	Q08AI8
**Semaphorin-4D**	52	97.5	14	Q92854
**Ninein**	51	245.2	39	Q8N4C6
**Unconventional myosin-VIIb**	51	243.6	13	Q6PIF6
**Endothelial zinc finger protein induced by tumor necrosis factor alpha**	51	56.2	14	Q9NQZ8
**Rab GDP dissociation inhibitor beta**	51	51.1	11	P50395
**Cytochrome P450 2B6**	50	56.5	9	P20813
**Zinc finger and SCAN domain-containing protein 5A**	50	56.9	7	Q9BUG6
**Gypsy retrotransposon integrase-like protein 1**	50	60.2	9	Q9NXP7
**HAUS augmin-like complex subunit 2**	50	27.1	4	Q9NVX0
**V-set and transmembrane domain-containing protein 2B**	50	30.4	8	A6NLU5
**Small nuclear ribonucleoprotein-associated protein N**	50	24.8	11	P63162
**Cyclin-dependent kinase 4 inhibitor C**	50	18.2	4	P42773
**UPF0704 protein C6orf165**	50	71.7	16	Q8IYR0
**Apolipoprotein L3**	50	44.6	11	O95236
**Putative uncharacterized protein ST20-AS1**	50	13.9	7	Q8NBB2
**N-chimaerin**	50	53.8	12	P15882
**Polyadenylate-binding protein 2**	50	32.8	5	Q86U42
**A-kinase anchor protein 1, mitochondrial**	50	98.3	8	Q92667

Protein name	Score	Mass	Matches	Access no.
**Alkyldihydroxyacetonephosphate synthase, peroxisomal**	49	73.7	9	O00116
**Smoothelin-like protein 2**	49	50.5	6	Q2TAL5
**Beta-defensin 136**	49	9.1	5	Q30KP8
**Protein C3orf33**	48	33.9	10	Q6P1S2
**GDNF family receptor alpha-2**	48	53.3	6	O00451
**Olfactory receptor 2M4**	48	35.7	4	Q96R27
**Serine/threonine-protein phosphatase 2A 55 kDa regulatory subunit B gamma isoform**	48	51.9	8	Q13362
**Tyrosine-protein kinase Fes/Fps**	48	93,5	7	P07332
**Hormone-sensitive lipase**	47	117.3	17	Q05469
**Caspase-4**	47	43.8	9.	P49662
**Podocin**	47	42.5	7	Q9NP85
**Kinesin heavy chain isoform 5C**	47	110	8	O60282
**Protein FAM3C**	46	24.9	11	Q92520
**WD repeat-containing protein 82**	45	35.5	7	Q6UXN9
**NADH dehydrogenase [ubiquinone] 1 alpha subcomplex subunit 2**	45	11	6	O43678
**Fibroblast growth factor-binding protein 3**	45	28.1	8	Q8TAT2
**Poly [ADP-ribose] polymerase 12**	45	80.5	8	Q9H0J9
**Cytochrome P450 2S1**	45	55.8	6	Q96SQ9
**Interferon beta**	44	22.6	7	P01574
**Guanine nucleotide-binding protein-like 3**	44	62.5	12	Q9BVP2
**One cut domain family member 3**	44	50.2	7	O60422
**Solute carrier family 25 member 40**	43	38.6	5	Q8TBP6
**CMRF35-like molecule 6**	43	25	4	Q08708

Protein name	Score	Mass	Matches	Access no.
**Zinc finger protein 232**	43	48.5	6	Q9UNY5
**Ropporin-1A**	43	24.2	5	Q9HAT0
**Mitochondrial import inner membrane translocase subunit Tim8 A**	43	11.2	3	O60220
**Paraneoplastic antigen Ma3**	43	53	5	Q9UL41
**Aladin**	42	60.4	8	Q9NRG9
**Histone H2A-Bbd type 1**	42	12.7	3	P0C5Y9
**Oncostatin-M**	42	28.8	7	P13725
**Interleukin-12 receptor subunit beta-2**	42	97.1		Q99665
**B melanoma antigen 4**	41	4.2	3	Q86Y28
**Mitochondrial import receptor subunit TOM5 homolog**	41	6.0	6	Q8N4H5
**LIM domain-containing protein ajuba**	41	58.7	6	Q96IF1
**Autoimmune regulator**	41	58.7	8	O43918
**Ras-related protein Rab-36**	40	36.8	8	O95755
**Zinc finger protein 525**	40	24.1	5	O95755
**Axin interactor, dorsalization-associated protein**	40	35.2	8	Q96BJ3
**Ig lambda-7 chain C region**	40	11.5	4	A0M8Q6
**Translation initiation factor eIF-2B subunit beta**	40	39.2	4	P49770
**Transmembrane protein 263**	40	11.7	3	Q8WUH6
**C-X-C chemokine receptor type 2**	39	41.2	4	P25025
**Collagen alpha-1(XXV) chain**	38	65.1	6	Q9BUG6
**Kunitz-type protease inhibitor 2**	37	29	4	O43291
**Zinc finger protein 674**	37	68.9	7	Q2M3X9
**Cystatin-C**	36	16	6	P1034

Protein name	Score	Mass	Matches	Access no.
**Calpain-2 catalytic subunit**	36	80.8	9	P17655
**Histone H2A type 1-A**	34	14.2	4	Q96QV6
**MICAL-like protein 2**	34	98.2	8	Q8IY33
**Thrombopoietin**	29	38	3	P40225
