# Supplementary material for: Tear film proteome in age-related macular degeneration
Source: Graefes Arch Clin Exp Ophthalmol. 2018 Apr 25;256(6):1127–39. doi: 10.1007/s00417-018-3984-y (PMC5956098; doi:10.1007/s00417-018-3984-y)
Supplement: Supplementary file 2 — (PDF 87 kb) [file 417_2018_3984_MOESM2_ESM.pdf]

Tear film proteome in age-related macular degeneration.

Mateusz Winiarczyk[1], Kai Kaarniranta[2], Stanisław Winiarczyk[3], Łukasz Adaszek[3], Dagmara Winiarczyk[3], Jerzy Mackiewicz*[1]

1. Department of Vitreoretinal Surgery, Medical University of Lublin
2. Department of Ophthalmology, University of Eastern Finland and Kuopio University Hospital, Kuopio, Finland
3. Department of Epizootiology, University of Life Sciences of Lublin

*jerzymackiewicz@umlub.pl, 20-079 Lublin, ul. Chmielna 1, Poland, tel./fax: +48 81 53 40 251

Supplementary table 2 - Wet AMD patients identified proteins

Nazwa białka	Score	Masa cząsteczkowa	Liczba dopasowań	Access no.
**Serum albumin**	271	71.3	17	P02768
**Serotransferrin**	221	79.3	30	P02787
**Lactotransferrin**	129	80.0	21	P02788
**Keratin, type I cytoskeletal 13**	123	49.9	20	P13646
**Cystatin - SN**	102	16.6	8	P01037
**Lipocalin-1**	94	19.4	7	P31025
**Keratin, type I cytoskeletal 19**	92	44.1	13	P08727
**Keratin, type II cytoskeletal 1**	90	66.2	19	P04264
**Cyclin-dependent kinase 4 inhibitor D**	85	17,7	12	P55273
**Cystatin-S**	73	16,5	6	P01036
**Keratin type I cytoskeletal 10**	70	59	13	P13645
**Nucleolar pre-ribosomal-associated protein 1**	70	256.5	24	O60287
**NADH dehydrogenase [ubiquinone] 1 alpha subcomplex subunit 2**	70	11	10	O43678
**28S ribosomal protein S22, mitochondrial**	69	41.4	6	P82650
**Telomerase reverse transcriptase**	67	128.6	11	O14746
**Caspase recruitment domain-containing protein 14**	67	114.9	11	Q9BXL6

Nazwa białka	Score	Masa cząsteczkowa	Liczba dopasowań	Access no.
**Pleckstrin homology domain-containing family A member 2**	67	47.9	8	Q9HB19
**Nutritionally-regulated adipose and cardiac enriched protein homolog**	67	18,4	7	Q8N912
**Suppressor of tumorigenicity 20 protein**	66	9.2	7	P40763
**Keratin, type I cytoskeletal 10**	66	59.0	17	P13645
**Testis-specific basic protein Y 2**	66	12,3	9	O14599
**BTB/POZ domain-containing protein KCTD1**	64	29.7	6	Q719H9
**Protein FAM183A**	64	16	7	A6NL82
**Calmodulin-regulated spectrin-associated protein 2**	64	169,2	23	Q08AD1
**HLA class I histocompatibility antigen, Cw-15 alpha chain**	64	41,3	9	Q07000
**Calponin-3**	63	36.6	10	Q15417
**Graves disease carrier protein**	63	36.5	5	P16260
**Collagen alpha-1(IX) chain**	63	92.4	6	P20849
**Transcriptional regulator ATRX**	63	284,9	54	P46100
**NADH dehydrogenase [ubiquinone] 1 alpha subcomplex assembly factor 4**	63	20,3	12	Q9P032
**Inactive ubiquitin carboxyl-terminal hydrolase 54**	63	189,6	20	Q6IE24
**Putative dehydrogenase/ reductase SDR family member 4-like 1**	62	30.8	6	P0CG22
**Retinal dehydrogenase 1**	62	55.5	13	P00352
**Rod cGMP-specific 3',5'-cyclic phosphodiesterase subunit beta**	62	99.5	14	P35913
**Phosphoinositide-interacting protein**	62	15.5	6	P0C851
**Lebercilin**	61	80.7	18	Q86VQ0
**Keratin type II cytoskeletal 1**	61	66.2	13	P04264

Nazwa białka	Score	Masa cząsteczkowa	Liczba dopasowań	Access no.
**Poly(rC)-binding protein 3**	61	39.7	4	P57721
**Prolactin-inducible protein**	61	16,8	8	P12273
**Uncharacterized protein ADAMTSL4-AS1**	61	4,3	4	Q5T5F5
**Probable ATP-dependent RNA helicase DDX47**	61	50,9	12	Q9H0S4
**Keratin, type II cytoskeletal 2 epidermal**	60	65.7	15	P35908
**TBC1 domain family member 9B**	60	141.6	14	Q66K14
**SRC kinase signaling inhibitor 1**	60	112,7	14	Q9C0H9
**Myosin-13**	60	224,6	35	Q9UKX3
**RING-box protein 2**	60	13,4	9	Q9UBF6
**Testis-expressed sequence 33 protein**	60	30,8	14	O43247
**Vacuolar fusion protein MON1 homolog B**	59	59.6	8	Q7L1V2
**Insulin-like growth factor-binding protein 3**	59	32.7	8	P17936
**Beta-defensin 107**	59	8.1	3	Q8IZN7
**Pseudokinase FAM20A**	59	62.1	9	Q96MK3
**Putative fatty acid-binding protein 5-like protein 3**	59	11,5	10	A8MUU1
**IgA-inducing protein homolog**	59	6,2	6	A6NJ69
**Zinc finger protein 585B**	59	90,6	15	Q52M93
**Protein deglycase DJ-1**	58	20.0	6	Q99497
**Keratin type II cytoskeletal 2 epidermal**	58	65.7	12	P35908
**MAP/microtubule affinity-regulating kinase 4**	58	83.3	17	Q96L34
**Bcl-2-like protein 10**	58	22.2	6	Q9HD36
**Annexin A3**	58	36,5	11	P12429
**Oculomedin**	58	5,3	6	Q9Y5M6
**Insulin gene enhancer protein ISL-2**	58	40,7	7	Q96A47
**Beta-defensin 125**	58	17,9	8	Q8N687
**Dystonin**	58	865,3	47	Q03001

Nazwa białka	Score	Masa cząsteczkowa	Liczba dopasowań	Access no.
**Protein FAM179A**	57	112.0	10	Q6ZUX3
**Keratin, type I cytoskeletal 9**	57	62.3	18	P35527
**LIM domain-containing protein ajuba**	57	58.7	9	Q96IF1
**Synapsin-3**	57	63.5	10	O14994
**Kunitz-type protease inhibitor 2**	57	29	7	O43291
**Zinc finger protein 473**	56	103.0	10	Q8WTR7
**Integrator complex subunit 6**	56	101.0	10	Q9UL03
**Heat shock factor-binding protein 1**	56	8.5	5	O75506
**Putative uncharacterized protein C6orf50**	56	12.6	5	Q9HD87
**Peroxiredoxin-6**	56	25.1	8	P30041
**Biogenesis of lysosome-related organelles complex 1 subunit 2**	56	16	7	Q6QNY1
**Molybdopterin synthase catalytic subunit**	56	21,2	8	O96007
**Serine/threonine-protein phosphatase 4 regulatory subunit 4**	56	100,5	22	Q6NUP7
**Protein asunder homolog**	55	81.1	11	Q9NVM9
**NADH dehydrogenase [ubiquinone] 1 alpha subcomplex subunit 9, mitochondrial**	55	42.7	19	Q16795
**Rho GTPase-activating protein 1**	55	11	50,5	P20936
**Immediate early response 3-interacting protein 1**	55	9	6	Q9Y5U9
**Pumilio homolog 3**	55	73,9	24	Q15397
**Parvalbumin alpha**	54	12.1	8	P20472
**Heterogeneous nuclear ribonucleoprotein M**	54	77,7	21	P52272
**Histatin-3**	54	6,1	5	P15516
**Spectrin beta chain, non-erythrocytic 4**	54	290	19	Q9H254

Nazwa białka	Score	Masa cząsteczkowa	Liczba dopasowań	Access no.
**Zinc finger and SCAN domain-containing protein 5A**	54	56,9	13	Q9BUG6
**Histone H1.4**	54	21,9	8	P10412
**Gamma-glutamylcyclotransferase**	54	21,2	4	O75223
**POTE ankyrin domain family member J**	54	118,7	16	P0CG39
**Hedgehog-interacting protein**	53	80.7	13	Q96QV1
**Coiled-coil domain-containing protein 25**	53	24.6	10	Q86WR0
**Interferon-induced protein with tetratricopeptide repeats 2**	53	55.3	10	P09913
**Fidgetin-like protein 1**	53	74,8	22	Q6PIW4
**Zinc finger protein 394**	53	65,5	15	Q53GI3
**Histone H2A type 2-B**	53	14	5	Q8IUE6
**Tudor domain-containing protein 3**	53	73,4	16	Q9H7E2
**Collagen alpha-1(XXI) chain**	53	100,1	13	Q96P44
**Ras-related protein Rab-4B**	53	23,9	7	P61018
**Bcl-2-related ovarian killer protein**	53	23,6	9	Q9UMX3
**Aurora kinase C**	52	35.9	7	Q9UQB9
**Alpha-N-acetylgalactosaminide alpha-2,6-sialyltransferase 6**	52	38,5	9	Q969X2
**Putative UPF0607 protein ENSP00000383144**	52	37,9	10	A8MX80
**Synaptotagmin-like protein 3**	52	69,7	13	Q4VX76
**Kinase suppressor of Ras 2**	52	108,9	17	Q6VAB6
**Shootin-1**	51	72.2	10	A0MZ66
**Protein CYR61**	51	44.2	10	O00622
**Ropporin-1A**	51	24.2	7	Q9HAT0
**Zinc finger protein 73**	51	39.6	7	O43830
**Transmembrane protein 263**	51	11,7	6	Q8WUH6
**A-kinase anchor protein C18orf42**	51	7,8	5	P0CW23

Nazwa białka	Score	Masa cząsteczkowa	Liczba dopasowań	Access no.
**Serpin B3**	51	44,6	10	P29508
**Gap junction beta-7 protein**	51	26,5	7	Q6PEY0
**Protein WFDC11**	51	10,8	6	Q8NEX6
**Elongator complex protein 6**	51	30,1	8	Q0PNE2
**Chondroitin sulfate N-acetylgalactosaminyltransferase 1**	51	61,2	16	Q8TDX6
**Methylmalonic aciduria type A protein, mitochondrial**	51	46,9	12	Q8IVH4
**Uncharacterized protein C6orf118**	51	54,3	14	Q5T5N4
**GTP-binding protein Di-Ras2**	51	22,8	8	Q96HU8
**NADH dehydrogenase [ubiquinone] 1 alpha subcomplex subunit 12**	50	17.1	5	Q9UI09
**Cyclin-G1**	50	34,7	15	P51959
**Protein UXT**	50	18,3	7	Q9UBK9
**Dynamin-3**	50	98,1	16	Q9UQ16
**Calcium/ calmodulin-dependent 3',5'-cyclic nucleotide phosphodiesterase 1A**	50	61,4	11	P54750
**Phosphoserine phosphatase**	50	25,2	7	P78330
**Collagen alpha-1(XIX) chain**	50	115,9	16	Q14993
**Phosphorylase b kinase regulatory subunit beta**	49	125,9	15	Q93100
**CGG triplet repeat-binding protein 1**	49	19,1	7	Q9UFW8
**HAUS augmin-like complex subunit 5**	49	72,3	11	O94927
**Zinc finger protein ZFP69B**	48	62,8	8	Q9UJL9
**Uncharacterized protein C2orf66**	47	13.4	4	Q6UXQ4
**Melanoma inhibitory activity protein 3**	47	214.3	14	Q5JRA6
**Apolipoprotein L6**	47	38,4	7	Q9BWW8
**Actin, cytoplasmic 1**	46	42.1	6	P60709
**Axonemal dynein light chain domain-containing protein 1**	45	118.6	8	Q5T1B0

Nazwa białka	Score	Masa cząsteczkowa	Liczba dopasowań	Access no.
**Inositol monophosphatase 1**	45	30.6	4	P29218
**Fibroblast growth factor 8**	44	26.7	5	P11362
**Interleukin-36 beta**	44	18,9	7	Q9NZH7
**TATA element modulatory factor**	41	123.3	9	P82094
**Sjoegren syndrome/ scleroderma autoantigen 1**	41	21.9	4	O60232
**Protein S100-A7A**	37	11,4	3	Q86SG5
**Rab GDP dissociation inhibitor beta**		51.1	7	Q9H0U4
