# Supplementary material for: Tear film proteome in age-related macular degeneration
Source: Graefes Arch Clin Exp Ophthalmol. 2018 Apr 25;256(6):1127–39. doi: 10.1007/s00417-018-3984-y (PMC5956098; doi:10.1007/s00417-018-3984-y)
Supplement: Supplementary file 3 — (PDF 62 kb) [file 417_2018_3984_MOESM3_ESM.pdf]

Tear film proteome in age-related macular degeneration.

Mateusz Winiarczyk[1], Kai Kaarniranta[2], Stanisław Winiarczyk[3], Łukasz Adaszek[3], Dagmara Winiarczyk[3], Jerzy Mackiewicz*[1]

1. Department of Vitreoretinal Surgery, Medical University of Lublin
2. Department of Ophthalmology, University of Eastern Finland and Kuopio University Hospital, Kuopio, Finland
3. Department of Epizootiology, University of Life Sciences of Lublin

*mateuszwiniarczyk@umlub.pl, 20-079 Lublin, ul. Chmielna 1, Poland, tel./fax: +48 81 53 40 251

Supplementary table 3 - Dry AMD patients identified proteins

Protein Name	Score	Mass	Matches	Access no.
Serum albumin	207	71.3	10	P02768
Zinc-alpha-2-glycoprotein	116	34.5	13	P25311
Bromodomain testis-specific protein	80	108.5	18	Q58F21
Unconventional myosin-Ih	78	120	19	Q8N1T3
Endothelial differentiation-related factor 1	78	16.4	8	O60869
Lipocalin-1	76	19.4	9	P31025
Protein SSX2	75	21.7	8	Q16385
Actin, cytoplasmic 1	74	42.1	9	P60709
Unconventional myosin-Ih	73	120	21	Q8N1T3
tetratricopeptide repeat protein	73	19.4	8	P31025
Integrator complex subunit 6	72	101	11	Q9UL03
A-kinase anchor protein 4	70	95.8	10	Q5JQC9
Integrator complex subunit 6	69	101	11	Q9UL03
Uncharacterized protein C1orf122	68	11.5	7	Q6ZSJ8
Grainyhead-like protein 3 homolog	67	71	8	Q8TE85
Integrator complex subunit 6	67	101	12	Q9UL03
Speedy protein A	66	39.8	9	Q6IBS0
Shootin-1	66	72.1	11	A0MZ66
Prolactin-inducible protein	66	16.8	7	P12273
Guanine nucleotide-binding protein subunit alpha-11	66	42.4	10	P29992
Prolactin-inducible protein	66	16.8	6	P12273
Integrator complex submit6	65	101	11	Q9UL03
Exocyst complex component 8	65	82.4	15	Q8IYI6

Protein Name	Score	Mass	Matches	Access no.
Desmin	65	53.6	9	P17661
SRC kinase signaling inhibitor	65	112.7	15	Q9C0H9
tRNA-dihydrouridine(20) synthase [NAD(P)+]-like	64	55.8	11	Q9NX74
AP-2 complex subunit alpha-1	64	108.6	7	O95782
Ferritin heavy polypeptide-like 17	64	21.4	8	Q9BXU8
Prolactin-inducible protein	64	16.8	9	P12273
Dynein heavy chain 17, axonemal	64	515.3	26	Q9UFH2
Dynein heavy chain 17, axonemal	63	515.3	32	Q9UFH2
Kinesin-like protein KIF28P	63	109.1	12	B7ZC32
Uncharacterized protein C10orf111	62	17.9	5	Q8N326
Neurofilament medium polypeptide	62	102.5	14	Q8NA69
Carbonyl reductase [NADPH] 3	62	31.2	8	O75828
Centromere/kinetochore protein zw10 homolog	62	89.6	19	O43264
Kinesin-like protein KIF28P	61	109.1	13	B7ZC32
Bloom syndrome protein	61	160.6	14	P54132
Speedy protein A	61	37.1	7	Q5MJ70
NADH dehydrogenase [ubiquinone] 1 alpha subcomplex subunit 10	61	41.1	10	O95299
Alkaline phosphatase, tissue-nonspecific isozyme	61	57.6	8	P05186
Putative uncharacterized protein C19orf81	61	22.7	5	C9J6K1
Rho GTPase-activating protein 24	61	84.8	14	Q8N264
Allograft inflammatory factor 1	60	16.7	6	P55008
Chondroitin sulfate N-acetylgalactosaminyltransferase 1	60	61.8	9	Q8TDX6
Hsp90 co-chaperone Cdc37-like 1	60	39.3	9	Q7L3B6
Uncharacterized protein C2orf61 homolog	60	27.7	5	Q9DAG5
NADH dehydrogenase [ubiquinone] 1 alpha subcomplex subunit 10, mitochondrial	60	41.1	10	O95299
A-kinase anchor protein 4	60	95.8	13	Q5JQC9
Tetratricopeptide repeat protein 34	60	61.8	9	A8MYJ7
Calpain-7	60	93.3	16	Q9Y6W3
Lipocalin-1	60	19.4	8	P31025
Putative uncharacterized protein SSBP3-AS1	59	11	4	Q7Z2R9
Cytoplasmic dynein 1 light intermediate chain 1	59	56.8	8	Q9Y6G9
Blood vessel epicardial substance	59	41.9	7	Q8NE79

Protein Name	Score	Mass	Matches	Access no.
Myc proto-oncogene protein	59	49.3	10	P01106
Endothelin-converting enzyme 1	59	87.9	8	P42892
Leucine-rich repeat-containing protein 16A	59	152.7	17	Q5VZK9
Alkyldihydroxyacetonephosphate synthase, peroxisomal	59	73.7	10	O00116
Trafficking protein particle complex subunit 1	59	16.9	8	Q9Y5R8
Chromobox protein homolog 2	58	56.4	8	Q14781
Kinesin-like protein KIF28P	58	109.1	12	B7ZC32
Ribosomal protein S6 kinase alpha-3	58	84	10	P51812
Methyltransferase-like protein 2B	58	44.1	9	Q6P1Q9
Aldo-keto reductase family 1 member B10	58	36.2	8	O60218
Chondroitin sulfate N-acetylgalactosaminyltransferase	58	61.8	9	Q8TDX6
Kinesin-like protein KIF28P	58	109.1	10	B7ZC32
Fibroblast growth factor 9	57	23.5	4	P31371
Rap guanine nucleotide exchange factor 2	57	168.2	17	Q9Y4G8
Cell differentiation protein RCD1 homolog	57	34	9	Q92600
Tetratricopeptide repeat protein 34	57	61.8	11	A8MYJ7
Sulfhydryl oxidase 2	57	78.2	11	Q6ZRP7
,	57	61.8	10	A8MYJ7
Multimerin	57	139.2	13	Q13201
cGMP-dependent protein kinase 2	57	88.1	10	Q13237
Zinc finger and SCAN domain-containing protein 31	57	48.2	5	Q96LW9
Bloom syndrome protein	56	160.6	15	P54132
Intraflagellar transport protein 140 homolog	56	166.9	16	Q96RY7
Uncharacterized protein C19orf45	56	58	7	Q8NA69
Sterile alpha motif domain-containing protein	56	19.4	6	Q5TGI4
Desmin	56	53.6	7	P17661
Glycine amidinotransferase, mitochondrial	56	48.9	9	P50440
Round spermatid basic protein 1	56	90.9	10	Q5VWQ0
Zona pellucida sperm-binding protein 2	56	83.5	9	Q05996
F-box/WD repeat-containing protein 1A	56	70.2	10	Q9Y297

Protein Name	Score	Mass	Matches	Access no.
Peroxiredoxin-1	56	22.3	8	Q06830
LINE-1 retrotransposable element ORF2 protein	56	150	10	O00370
WD repeat-containing and planar cell polarity effector protein fritz homolog	56	86.2	11	O95876
Desmin	55	53.6	9	P17661
Zinc finger and SCAN domain-containing protein 5A	55	56.9	9	Q9BUG6
C-type lectin domain family 2 member L	55	24.4	5	P0C7M8
Pyruvate kinase PKM	55	58.5	5	P14618
Nucleoside diphosphate-linked moiety X motif 6	55	36	9	P53370
Elongation factor Ts, mitochondrial	55	35.7	6	P43897
Rab GTPase-binding effector protein 2	54	64	9	Q9H5N1
ZN624_HUMAN	54	102.5	15	Q9P2J8
Lipocalin-1	54	19.4	6	P31025
Armadillo repeat-containing protein 4	53	117.1	12	Q5T2S8
Aldo-keto reductase family 1 member B10	53	36.2	7	O60218
Neurofilament medium polypeptide	53	58	9	Q8NA69
5'-AMP-activated protein kinase subunit gamma-2	53	63.2	8	Q9UGJ0
Zinc finger MYND domain-containing protein 15	53	82.7	12	Q9H091
5'-AMP-activated protein kinase subunit gamma-2	53	63.2	8	Q9UGJ0
Desmin	53	53.6	12	P17661
Vitamin D-binding protein	53	54.5	8	P02774
Tetratricopeptide repeat protein	52	61.8	9	A8MYJ7
Uncharacterized protein C19orf45	52	58	6	Q8NA69
Bloom syndrome protein	52	160.6	13	P54132
Zinc finger protein 780A	52	76.6	10	O75290
Histatin-3	52	6.1	5	P15516
Tyrosine-protein kinase Fer	51	95.2	13	P16591
Tetratricopeptide repeat protein	51	61.8	7	A8MYJ7
Integrator complex subunit 6	51	101	8	Q9UL03
Protein S100-A7A	51	11.4	5	Q86SG5
WD repeat-containing and planar cell polarity effector protein fritz homolog	50	86.2	8	O95876

Protein Name	Score	Mass	Matches	Access no.
39S ribosomal protein L49, mitochondrial	50	19.2	5	Q13405
B-cell antigen receptor complex-associated protein beta chain	50	26.3	5	P40259
Translation machinery-associated protein 16	50	24.1	6	Q96EY4
Dual specificity protein kinase CLK3	50	47.3	8	P49761
Probable protein phosphatase 1N	50	46.9	7	Q8N819
Ly6/PLAUR domain-containing protein 4	49	27.5	6	Q6UWN0
Nucleoporin Nup43	49	42.6	5	Q8NFH3
Ubiquinone biosynthesis O-methyltransferase, mitochondrial	48	41.5	4	Q9NZJ6
Ciliogenesis-associated TTC17-interacting protein	48	44.3	5	Q7Z7H3
Integrator complex subunit 6	48	101	8	Q9UL03
Thrombopoietin	47	38	6	P40225
Rab GDP dissociation inhibitor beta	46	51.1	7	P50395
WW domain-binding protein 5	46	12.8	6	Q9UHQ7
Multimerin-1	45	139.2	7	Q13201
Ubiquinone biosynthesis O-methyltransferase, mitochondrial	45	41.5	3	Q9NZJ6
Ubiquitin/ISG15-conjugating enzyme E2 L6	45	17.9	4	O14933
Myosin light chain 6B	45	22.9	5	O14933
Ganglioside-induced differentiation-associated protein 2	45	56.6	7	Q9NXN4
Putative uncharacterized protein encoded by COL5A1-AS1	44	6.6	4	Q5SY13
DNA oxidative demethylase ALKBH1	44	44.2	4	Q13686
Rab GDP dissociation inhibitor beta	43	51.1	8	P50395
DNA repair protein XRCC3	41	38.3		O43542
Ubiquinone biosynthesis O-methyltransferase	40	41.5	4	Q9NZJ6
Glutamate receptor 2	40	99.4	7	P42262
Cystatin-S	38	16.5	5	P01036
5'-AMP-activated protein kinase subunit gamma-2		63.2	7	Q9UGJ0
